# Acquired Von Willebrand's Syndrome in Systemic Lupus Erythematosus

**DOI:** 10.1155/2014/208597

**Published:** 2014-12-07

**Authors:** Sara Taveras Alam, Karenza Alexis, Ashwin Sridharan, Marianna Strakhan, Tarek Elrafei, Richard J. Gralla, Louis J. Reed

**Affiliations:** ^1^Department of Internal Medicine, Albert Einstein College of Medicine, Jacobi Medical Center, Bronx, NY 10461, USA; ^2^Division of Hematology and Oncology, Albert Einstein College of Medicine, Jacobi Medical Center and Montefiore Medical Center, Bronx, NY 10461, USA

## Abstract

Acquired von Willebrand syndrome (AVWS) is an uncommon, underdiagnosed, and heterogeneous disease which is increasingly recognized as a cause of bleeding diatheses. Systemic lupus erythematosus (SLE) is an infrequent cause of AVWS. Herein, we report a case of AVWS diagnosed during the initial presentation of SLE in a previously healthy young man with no family history of bleeding diathesis who presented with worsening epistaxis, gastrointestinal bleeding, and anasarca. He was found to have severe anemia and prolonged activated partial thromboplastin time (aPTT) with severely decreased levels of von Willebrand factor (VWF) measurements in addition to markedly decreased factor VIII levels. Further evaluation revealed nephrotic syndrome and interstitial lung disease due to SLE. He initially received combination therapy with intravenous immunoglobulin (IVIG) and von Willebrand factor/factor VIII concentrates without significant improvement. Treatment with steroids, cyclophosphamide, and rituximab was followed by clinical improvement evidenced by cessation of bleeding. The short follow-up did not allow us to definitely prove the therapeutic effect of immunosuppressive treatment on AVWS in SLE patients. This case adds to the literature supporting the relationship between AVWS and SLE and highlights the importance of combination therapy in the treatment of severe AVWS as well as the role of IVIG, cyclophosphamide, and rituximab in AVWS associated with SLE.

## 1. Introduction

Acquired von Willebrand syndrome (AVWS) is a rare but increasingly recognized cause of bleeding diathesis. It should be suspected in patients with mucocutaneous bleeding without a prior personal or family history of bleeding disorders and an isolated elevated activated partial thromboplastin time. Systemic lupus erythematosus (SLE) is an infrequent cause of AVWS, but associations between these two diseases have been documented and described in the literature [1–18]. Herein, we present a case of severe AVWS diagnosed during the initial presentation of SLE in a previously healthy young man.

## 2. Case Report

A 26-year-old Mexican male with no significant past medical history other than a childhood appendectomy presented to the emergency department after several weeks of worsening epistaxis. He also admitted to have easy bruising, 1-2 episodes of tarry stools, lower extremity swelling, and increased abdominal girth during this time, as well as fatigue and decreased exercise tolerance.

He denied prior bleeding or family history of bleeding disorders or autoimmune conditions.

Physical exam was significant for pallor, periorbital edema, dried blood in the vestibule of right nostril and red blood to the left nostril, and petechiae to the soft palate without notable bleeding in the oropharynx. Also noted on exam were elevated jugular venous pressure, tachycardia at 110 beats per minute, decreased breath sounds on the right lung base, abdominal distension with dullness to percussion, and bilateral 2+ pitting lower extremity edema up to the thighs. Stool guaiac performed in the emergency department was positive.

On initial laboratory evaluation, the patient was found to have severe normocytic anemia with hemoglobin (Hb) of 4.6 g/dL (MCV 87 fL, RDW 18.9%, reticulocytes 2.79%) and a prolonged activated partial thromboplastin time (aPTT) at 50.6 sec with a normal prothrombin time. Initial laboratory testing was also remarkable for thrombocytopenia at 112/nL and severe hypoalbuminemia at 1.1 g/dL; see Table 1 for reference values. Fibrinogen levels were within normal limits and LDH was only mildly elevated at 290 U/L (normal range 140–280 U/L). Peripheral blood smear was remarkable only for mild thrombocytopenia.

Four units of packed red blood cells (PRBCs) were administered with subsequent Hb increase to only 6.7 g/dL, corroborating significant active bleeding. Two units of fresh frozen plasma (FFP) were given without improvement in his coagulation profile and hematological opinion was requested to further evaluate coagulopathy.

Subsequent testing demonstrated normal factor IX and factor XI levels but factor VIII was markedly decreased at 2% (normal range 60–150%) and von Willebrand factor activity/Ristocetin cofactor was undetectable (0%, normal range 50–150%). Von Willebrand factor antigen (VWF Ag) was also low at 8% (normal range 50–217%). Acquired von Willebrand's syndrome was diagnosed. Inherited type III VWD was deemed unlikely given history of appendectomy without bleeding complications. Given clinical suspicion of AVWS, he received loading dose of VWF containing concentrate (Humate-P, which was given at 40 U/kg) along with intravenous immune globulin (IVIG) at 400 mg/kg with a plan to give one dose a day over three days. Mixing study was unfortunately not performed prior to FFP administration, which did correct aPTT from 41 to 31. Yet VWF activity and factor VIII levels continued to be low despite treatment with VWF containing concentrate, supporting the presence of a factor VIII inhibitor. The patient continued to have some epistaxis after initial VWF containing concentrate and IVIG. Consequently, IVIG was increased to 1 g/kg/d on day 2 of treatment and VWF containing concentrate dosing was also increased with eventual bleeding control. VWF activity persisted at 0% despite three days of IVIG and ten doses of VWF containing concentrate.

Hypoalbuminemia and anasarca in the absence of liver disease prompted workup for nephrotic syndrome. The patient was found to have near nephrotic range proteinuria (Prot/Cr ratio 2477 mg/g) and dyslipidemia (HDL 9.9 mg/dL). Abdominal US confirmed mild-to-moderate ascites and mild-to-moderate increased renal cortical echogenicity bilaterally as well as subcapsular edema of both kidneys. All of these findings were in fact consistent with nephrotic syndrome.

Renal biopsy was deferred due to bleeding diathesis. Hepatitis and syphilis serologies as well as rapid oral HIV antibody test were negative. Serum protein electrophoresis (SPEP) and urine protein electrophoresis (UPEP) did not show monoclonal bands; antineutrophil cytoplasmic antibodies (pANCA and cANCA) and glomerular basement membrane antibodies (GBM Ab) were negative. Complement levels were markedly decreased (C3 < 30 mg/dL and C4 < 6 mg/dL). Antinuclear antibody panel was significant for a positive dsDNA Ab (344 IU/mL) and low positive histone Ab (142 IU/mL); however the patient had not been on medications prior to admission to suggest drug induced lupus. Antinuclear antibody was positive at 1 : 320, with a homogeneous pattern. Cardiolipin IgM/IgG and lupus anticoagulant were negative. Rheumatology consultation recommended initiation of stress dose steroids as well as obtaining high resolution chest computerized tomography, which demonstrated central ground glass opacities.

The patient met criteria for systemic lupus erythematosus (SLE) based on Lupus International Collaborating Clinics Classification criteria due to positive ANA titer, positive anti-dsDNA, low C3 and C4 levels, and thrombocytopenia and renal involvement.

In total, the patient received 5 units of PRBCs, 2 units of FFP, 15 doses of VWF containing concentrate, IVIG for 3 days (30 g IVSS on day 1, 75 g IVSS on day 2, and 45 g IVSS on day 3 for 2 g/kg total dose), and pulse steroids (solumedrol 1 g per day for 3 days and then solumedrol 80 mg per day for 2 days) that were switched to maintenance prednisone 60 mg po daily thereafter. He also received a single dose of desmopressin 22.5 mcg without improvement in his laboratory values. Nephrotic syndrome was managed with diuretics and antihypertensives including ACE inhibitors. On day 8 of treatment, he was given one dose of IV cyclophosphamide (750 mg IVSS) and one dose of rituximab (375 mg/kg IVSS) with subsequent clinical improvement. The patient was discharged in stable condition and on outpatient follow-up reported only minimal self-remitting epistaxis since his discharge.

The patient received a total of four weekly doses of rituximab 375 mg/m^2^. One month after presentation, anasarca and hypoalbuminemia improved (albumin 2.5 g/dL), Hb had remained stable at 8.1 g/dL without signs of bleeding, and thrombocytopenia resolved. VW factor activity however remained at 0%. He has since then been lost to follow-up, limiting further assessment of his response.

## 3. Discussion

AVWS is an uncommon, underdiagnosed, and heterogeneous disease which is more frequently being recognized as a cause of bleeding diatheses [1, 19–22]. Most cases present with mild-to-moderate mucocutaneous bleeding in the absence of a personal or family history of bleeding. Laboratory findings are similar to those of congenital von Willebrand disease (VWD), frequently with a prolonged bleeding time and low levels of von Willebrand factor measurements (VWFAg, functional assays and/or abnormal VWF multimers). Some cases have borderline to normal values of VWF functional assays, with a ratio to VWF Ag less than 0.7 indicating inhibitory antibodies or decrease in high molecular weight multimers, whereas under normal conditions VWF : RCo/Ag ratio or VWF : CBA/Ag ratio should be close to one [18]. Factor VIII may be decreased and activated partial thromboplastin time may be prolonged, as was the case in our patient.

Literature review suggests that, with the exception of patients with AVWS associated with hypothyroidism, low levels of VWF in AVWS are due to accelerated removal of the protein from the plasma via one of five described mechanisms: (1) specific autoantibodies that inactivate or increase the clearance of FVIII/VWF; (2) nonspecific antibodies that form circulating complexes with VWF, which are then cleared by Fc-bearing cells; (3) absorption of VWF onto malignant cell clones or platelets; (4) increased proteolytic degradation of VWF; or (5) loss of VWF multimers in high shear stress conditions [18, 23–27]. Only a minority of cases are due to factor VIII/von Willebrand factor-inhibiting activities [28], which alongside increased clearance due to antibody complexes represent the plausible explanations for AVWS in patients with SLE.

An international registry of 186 collected cases published in 2000 reported that the most common disorders associated with acquired von Willebrand syndrome were, in order of decreasing frequency: lymphoproliferative, cardiovascular, and myeloproliferative disease, as well as neoplasias. Other reported underlying conditions include drugs, infectious diseases, uremia, and hypothyroidism. Immune-mediated underlying causes are less common and only accounted for 2% of registry cases and 6% of cases found upon literature review at that time [25]. A follow-up publication four years later recognized that there may have been a bias towards disproportionately fewer cases of AVWS reported in association with immunologic disorders in the registry due to the low participation of immunologists or rheumatologists [26]. Besides SLE, other immunologic disorders that have been reported to be associated with AVWS include mixed connective tissue disease, scleroderma, Sjogren's syndrome, antiphospholipid syndrome, and graft versus host disease [12, 17, 29, 30].

The association between SLE and AVWS was first described in a 12-year-old boy with lupus nephritis in 1968 [14]. To this date, on our review of the literature, close to 700 AVWS cases have been reported [18, 25, 31, 32] and approximately twenty cases have been associated with SLE or lupus-like connective tissue disorder (see Table 2). Michiels has reported the presentation and course of all cases in the English language literature of AVWS and systemic lupus erythematosus up until 2001 [33]. In total, 12 cases were described [3, 5, 7, 8, 12–17]. Since 2001, there have been at least nine other reported cases of AVWS associated with SLE, including one patient with SLE in a retrospective single-center cohort study of 35 patients diagnosed with AVWS at Hannover Medical School [1, 2, 4, 6, 10, 11, 18, 34]. Most cases present at the time a patient is diagnosed with SLE or in patients already diagnosed with SLE. However, it has also been reported as a “herald” syndrome in a patient who later developed this disease [1, 11, 14, 16]. This raises the question of whether the published cases with lupus-like features that did not meet SLE diagnosis criteria in full [5, 7, 9] would have developed florid SLE with time if the disease was not treated or if there had been longer follow-up.

It is important to note that permanent reversal of the bleeding disorder may occur with treatment of the associated disease. Therefore, a reasonable workup for such associated diseases should be undertaken in any patient with AVWS [35]. In fact, bleeding symptoms due to AVWS can establish the need for treating an underlying disorder that otherwise may not have been treated, like monoclonal gammopathy of unknown significance [18].

In addition to aiming treatment of AVWS at the underlying cause or associated disease when possible, efforts should also focus on controlling and preventing bleeding episodes. Due to the heterogeneity of AVWS, no single drug is effective for all cases [36]. Accordingly, treatment usually involves a customized combination of available options: DDAVP, FVIII/VWF concentrates, high dose intravenous immunoglobulin (IVIG), plasmapheresis, corticosteroids, and immunosuppressive or chemotherapeutic agents. Recombinant factor VIIa has also been used in resistant cases [4] and antifibrinolytics have been used as adjunct therapies in cases associated with bleeding in areas of increased fibrinolysis. In AVWS associated with SLE, response to DDAVP and VWF/FVII is poor whereas IVIG has been effective and corticosteroids may provide cure [37]. Chemotherapeutic agents such as cyclophosphamide and rituximab have been of use in a few cases [10, 16, 22, 38, 39]; see Table 3.

The effect of desmopressin in AVWS is of lesser magnitude compared to congenital VWD because the increase in the VWF-FVIII complex is short-lived due to autoantibodies or increased clearance [40]. VWF containing concentrates, despite being more effective than DDAVP for AVWS, may also have a short half-life in patients with inhibitors [37]. Large doses may be required to overwhelm the autoantibody against exogenous VWF provided by the concentrates or to counterbalance the accelerated clearance of the VWF-FVIII complex [27].

IVIG has been described to temporarily normalize factor VIIIc and VWF levels [5, 16], especially in cases characterized by anti-FVIII/VWF inhibitors [40]. The underlying mechanism has been proposed to be related to immunoglobulin mediated elimination of circulating immune complexes, anti-idiotype effect, direct inhibitory effect on the cells producing the anti-VWF antibody, or blockage of reticuloendothelial Fc-receptors preventing phagocytosis and clearance of antibody-antigen complexes [41, 42]. Although IVIG can produce a more sustained response than DDAVP or factor concentrates, it requires 24–48 hours to normalize plasma VWF-FVIII activity; thus DDAVP or VWF-FVIII concentrates can be given together with IVIG to achieve hemostasis immediately in cases of emergency bleeding [22, 25]. Repeated doses of IVIG every 21 days can produce consistent responses, as has been shown in patients with IgG-MGUS [41, 43].

Steroids have been shown to be effective in most reported cases of AVWS associated with SLE, including the first case ever described [1, 3, 6–8, 11, 13–17, 34]. In addition, adjuvant single doses of cyclophosphamide (700 mg/m^2^) have assisted in correcting factor VIII and VWF levels to reference values in a case with incomplete response to steroids [16].

Rituximab has known efficacy in treating acquired factor VIII inhibitors [44] and, although it has been ineffective in AVWS associated with MGUS, it has been shown to be effective for AVWS associated with B cell lymphoma or multiple myeloma [22, 38, 39]. It has also been reported to be effective in AVWS associated with juvenile SLE, following failure to respond to treatment with first and second line therapies [10]. The role of rituximab in AVWS may go beyond its part in treating the underlying disease. In cases related to antibody induced clearance of factor VIII/VWF, its effectiveness is possibly due to its anti-CD20 properties, specifically depleting B cells, and possible blockage of the macrophage Fc-receptor function by rituximab-opsonized B cells [45].

Our patient initially received combination therapy with IVIG and VWF-FVIII concentrates without significant improvement. He then received steroids, cyclophosphamide, and rituximab with clinical improvement. Interestingly however, his von Willebrand factor measurements were not documented to have been corrected despite his clinical improvement. This may have been related to limited follow-up after treatment.

## 4. Summary

A case of severe AVWS diagnosed concurrently with newly diagnosed SLE in a previously healthy young man is described. This case supports the previously described intimate association between AVWS and SLE, the need for combination therapy, and the fact that reversal of the bleeding disorder may occur with treatment of the associated disease. In any patient with AVWS, in addition to treatment of the bleeding diathesis, a search for an underlying associated disease should be undertaken. Treatment should be primarily aimed at the underlying disorder, if found.

In our review of the literature, only approximately 20 cases of the close to 700 reported cases of AVWS have been associated with SLE. AVWS may present concurrently with SLE or in a patient already diagnosed with SLE and has also been described as a “herald syndrome” in patients who later develop lupus. The pathophysiology of this association is likely related to increased clearance of factor VIII/von Willebrand factor via antibodies or factor VIII/von Willebrand factor-inhibiting activities. In AVWS associated with SLE, response to DDAVP and VWF/FVIII is poor, whereas IVIG has been effective and corticosteroids may provide cure. Chemotherapeutic agents such as cyclophosphamide and rituximab may be required after failure to respond to other therapies.

## Figures and Tables

**Table 1:** Laboratory data of patient.

	Initial	Final^∧^	Reference values
WBC	11.0/nL	7.8/nL	3.9–10.6/nL
Hb	4.6 g/dL	8.1 g/dL	13.5–17.5 g/dL
Hct	14.20%	25.80%	41–53%
Plt	112/nL^*^	450/nL	150–440/nL
INR	1.06	1.00	0.9–1.2
aPTT	50.6 sec	43.7 sec	20.1–31.2 sec
Fibrinogen	308 mg/dL	NT	200–400 mg/dL
VWF : Ag	8%	10%	5–217%
VWF : RCo	0%	0%	50–150%
FVIII : C	2%	6%	60–150%
Cr	0.9 mg/dL	0.8 mg/dL	0.1–1.5 mg/dL
Albumin	1.1 g/dL	2.5 g/dL	3.5–5.5 g/dL
Protein/Cr ratio	2477 mg/g	NT	≤84 mg/g
ANA	1 : 320, homogeneous	NT	<1 : 80
Anti-dsDNA	344 IU/mL	NT	≤99 IU/mL
C3	<30 mg/dL	NT	90–180 mg/dL
C4	<6 mg/dL	NT	10–40 mg/dL
CRP	5.7 mg/L	NT	0–5.0 mg/L
ESR	100 mm/h	NT	0–15 mm/h
Reticulocytes	3.21%	NT	0.5–2%
LDH	290 U/L	NT	100–210 U/L
Haptoglobin	219 mg/dL	NT	43–212 mg/dL
Total bilirubin	0.1 mg/dL	NT	0.1–1.2 mg/dL
AST	19 U/L	NT	1–40 U/L
ALT	9 U/L	NT	1–40 U/L
ALP	49 U/L	NT	30–115 U/L
Direct Coombs test	Negative	NT	Negative
TSH	10.773 mIU/L	NT	0.47–6.90 mIU/L
Free T4	1.19 ng/dL	NT	0.74–2 ng/dL

WBC: white blood cell count; Hb: hemoglobin; Hct: hematocrit; Plt: platelet count; INR: international normalized ration; aPTT: activated partial thromboplastin time; VWF : Ag: von Willebrand factor antigen; VWF : RCo: von Willebrand factor ristocetin cofactor; FVIII : C: factor VIII procoagulant activity; Cr: creatinine; NT: not tested; ANA: antinuclear antibodies; anti-dsDNA: anti-double stranded DNA antibodies; C3: complement component 3; C4: complement component 4; CRP: C reactive protein; ESR: erythrocyte sedimentation rate; LDH: lactate dehydrogenase; AST: aspartate aminotransferase; ALT: alanine aminotransferase; ALP: alkaline phosphatase; TSH: thyroid stimulating hormone.

^∧^At last follow-up.

^*^Plt decreased to 86/nL on the 4th day of admission.

**Table 2:** AVWS in cases associated with SLE or lupus-like serology published to present date.

Author (Year)	Title
Simone et al. (1968) [14]	Acquired von Willebrand's syndrome in systemic lupus erythematosus

Ingram et al. (1971) [8]	Four cases of acquired von Willebrand's syndrome^*^

Poole-Wilson (1972) [13]	Acquired von Willebrand's syndrome and systemic lupus erythematosus

Gazengel et al. (1978) [3]	Antibody-induced von Willebrand syndrome: inhibition of VIII VWF and VIII AGN with sparing of VIII AHF by the autoantibody

Pizzuto et al. (1980) [12]	Acquired von Willebrand's syndrome during autoimmune disorder

Yoshida et al. (1988) [17]	Development of acquired von Willebrand's disease after mixed connective tissue disease

Igarashi et al. (1989) [7]	Acquired von Willebrand's syndrome with lupus-like serology

Soff and Green (1993) [15]	Autoantibody to von Willebrand factor in systemic lupus erythematosus

Hanley et al. (1994) [5]	Acquired von Willebrand's syndrome in association with a lupus-like anticoagulant corrected by intravenous immunoglobulin

Jackson et al. (1995) [9]	Puerperal acquired factor VIII inhibitor causing a von Willebrand-like syndrome in a patient with anti-DNA antibodies

Viallard et al. (1999) [16]	Three cases of acquired von Willebrand disease associated with systemic lupus erythematosus^***^

Niiya et al. (2002) [11]	Acquired type 3-like von Willebrand syndrome preceded full-blown systemic lupus erythematosus

Casonato et al. (2002) [2]	Lack of multimer organization of von Willebrand factor in an acquired von Willebrand syndrome

Tiede et al. (2008) [18]	Diagnostic workup of patients with acquired von Willebrand syndrome: a retrospective single-center cohort study^**^

Hong et al. (2008) [6]	Systemic lupus erythematosus complicated by acquired von Willebrand's syndrome

Kasatkar et al. (2013) [1]	Acquired von Willebrand syndrome: a rare disorder of heterogeneous etiology^***^

Jimenez et al. (2013) [10]	Rituximab effectiveness in a patient with juvenile systemic lupus erythematosus complicated with acquired Von Willebrand syndrome

Hami et al. (2014) [4]	Acquired von Willebrand syndrome in a male with systemic lupus erythematosus presented with mucocutaneous bleeding and was treated with rFVIIa

^*^One of four cases presented in this paper was associated with SLE.

^**^Three cases are presented, all of them associated with SLE.

^***^One case of those studied had SLE.

**Table 3:** Therapeutic options in acquired von Willebrand syndrome associated with SLE.

Therapeutic option	Dose	Comment
Desmopressin	0.3 *μ*g/kg over 30 minutes once or twice daily	Limited response, use in combination with IVIG

VWF containing concentrates	30–100 VWF : RCo units/kg	Dose depending on the patient's residual activity, severity of bleeding, and presence of inhibitors; also limited response, use in combination with IVIG

Recombinant factor VIIa	90 *μ*g/kg (range: 40–150 *μ*g/kg) every 2–6 hours	For a median of 3 doses (range 1–54 doses)

IVIG	1 g/kg/d for 2 days	May repeat a single high-dose 1 g/kg/d every 21 days; use of desmopressin and VWF containing concentrates is recommended initially for bleeding control as response is not immediate

Steroids	Prednisone 30–40 mg/d or 1-2 mg/kg per day	It treats underlying cause

Cyclophosphamide	700 mg/m^2^	It treats underlying cause; single doses have been effective but treatment may be repeated monthly

Rituximab	375 mg/m^2^ weekly for 2–4 weeks	Has been followed by doses every 90 days as long-term management in AVWS associated with B cell lymphoma

IVIG: intravenous immunoglobulin.
